# Three-port approach *vs* standard laparoscopic radical cystectomy with an ileal conduit: a single-centre retrospective study

**DOI:** 10.1186/s12894-021-00920-6

**Published:** 2021-11-15

**Authors:** Zhouting Tuo, Ying Zhang, Jinyou Wang, Huan Zhou, Youlu Lu, Xin Wang, Chao Yang, Dexin Yu, Liangkuan Bi

**Affiliations:** Department of Urology, The Second Affiliated Hospital of Anhui Medical University, NO. 678 Furong Road, Hefei, 230032 China

**Keywords:** Three-port, Laparoscopic radical cystectomy, Bladder cancer, Ileal conduit

## Abstract

**Background:**

This study aimed to evaluate the effect of the three-port approach and conventional five-port laparoscopic radical cystectomy (LRC) with an ileal conduit.

**Methods:**

Eighty-four patients, who were diagnosed with high-risk non-muscle-invasive and muscle-invasive bladder carcinoma and underwent LRC with an ileal conduit between January 2018 and April 2020, were retrospectively evaluated. Thirty and fifty-four patients respectively underwent the three-port approach and five-port LRC. Clinical characteristics, pathological data, perioperative outcomes, and follow-up data were analysed.

**Results:**

There were no differences in perioperatively surgical outcome, including pathology type, prostate adenocarcinoma incidence, tumour staging, and postoperative creatinine levels between the two groups. The operative time (271.3 ± 24.03 vs. 279.57 ± 48.47 min, *P* = 0.299), estimated blood loss (65 vs. 90 mL, *P* = 0.352), time to passage of flatus (8 vs. 10 days, *P* = 0.084), and duration of hospitalisation post-surgery (11 vs. 12 days, *P* = 0.922) were no clear difference between both groups. Compared with the five-port group, the three-port LRC group was related to lower inpatient costs (12 453 vs. 14 134 $, *P* = 0.021). Our follow-up results indicated that the rate of postoperative complications, 90-day mortality, and the oncological outcome did not show meaningful differences between these two groups.

**Conclusions:**

Three-port LRC with an ileal conduit is technically safe and feasible for the treatment of bladder cancer. On comparing the three-port LRC with the five-port LRC, our technique does not increase the rate of short-term and long-term complications and tumour recurrence, but the treatment costs of the former were reduced.

## Background

Radical cystectomy (RC) and urinary diversion remain standard procedures for high-risk non-muscle-invasive and muscle-invasive bladder carcinoma [1]. However, RC is one of the most challenging urologic surgeries because of its postoperative complications and high mortality. Nevertheless, treatment of bladder cancer currently achieves satisfactory therapeutic results through open radical cystectomy (ORC), laparoscopic radical cystectomy (LRC), and robot-assisted radical cystectomy (RARC) [2, 3].

The number of surgeons performing RARC has gradually increased since its first reported use [4]. There is evidence that RARC has the advantages of reducing the complication rate and enhancing postoperative recovery, and its therapeutic effect is equivalent to that of ORC [3, 5, 6]. Although many bladder cancer patients undergo RARC, the treatment costs remain high [7]. Urologists face this problem in many developing countries. Especially in China, robotic systems only exist in large medical centres, thus limiting RARC availability. Similar situations exist in other developing countries [8]; therefore, traditional LRC is still the mainstay of treatment for bladder cancer.

Traditional LRC usually requires five or four ports and has been popular because of its low cost and satisfactory postoperative outcomes [9]. However, a higher number of ports leads to the requirement of more surgeons and increases the possibility of port site-related complications. With the accumulation of surgical experience using laparoscopy, we devised a modified three-port technique for minimising the number of ports, which could be performed by a single surgeon and one scope assistant [10]. The initial results showed that our modified method was feasible and repeatable. In this investigation, we compared the clinical data of patients who underwent the three-port approach and conventional five-port LRC with an ileal conduit, including surgical, oncological, and postoperative outcomes.

## Methods

### Patients

Patients treated with three-port approach LRC and ileal conduit urinary diversion at our centre from January 2018 to April 2020 were reviewed and included in our study. Their clinical data were identified from the central database. Patients with severe obesity (body mass index [BMI] ≥ 35 kg/m^2^), distant metastases, poor renal function, severe liver insufficiency, active enteritis, and positive urethral margins were excluded. For comparison with these patients, other patients who underwent conventional five-port LRC and ileal conduit surgery at our centre during the same period were reviewed and matched to the study group by demographic characteristics (e.g. age, sex, BMI, and clinical tumour stage).

### Surgical technique

**Fig. 1:**
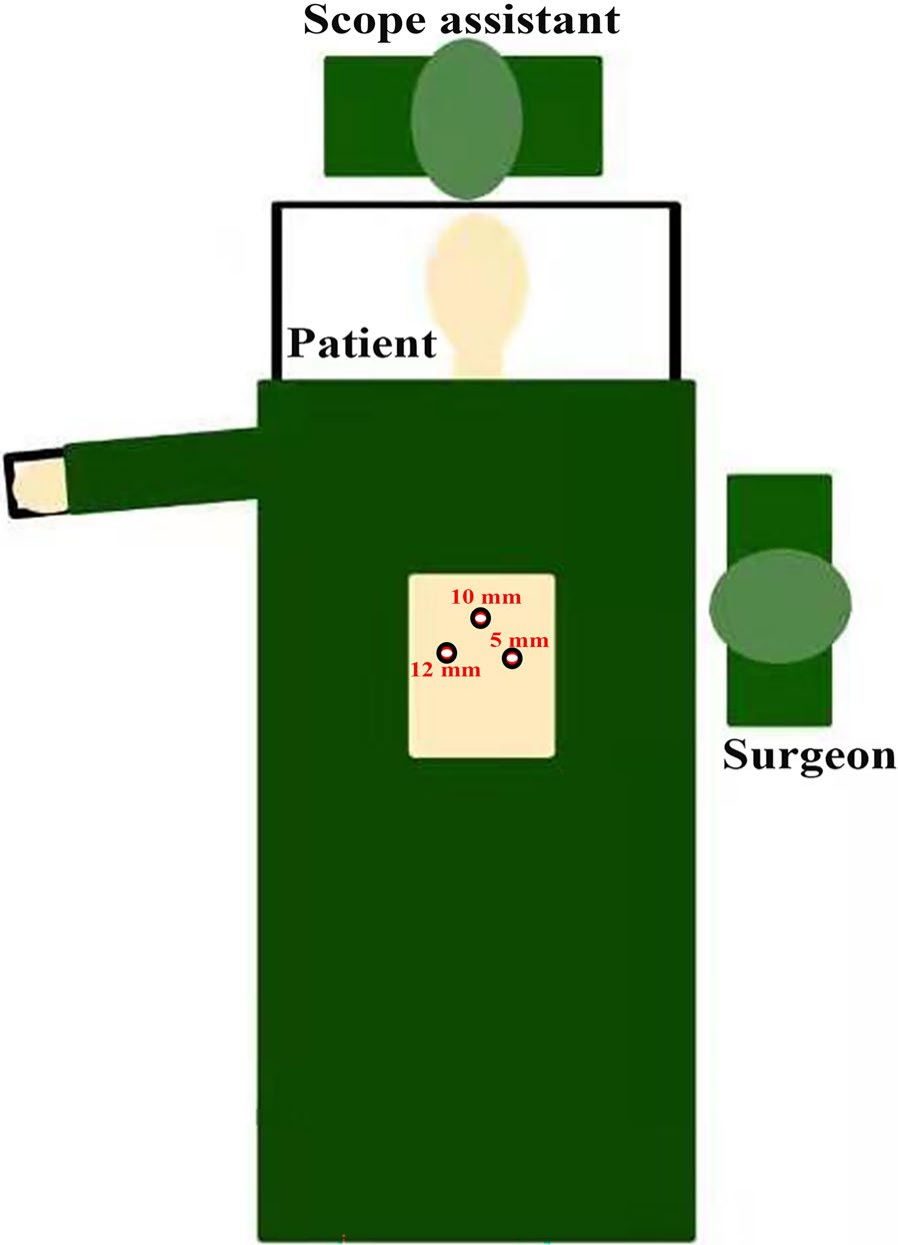
Schematic diagram of three-port LRC. The surgeon stood on the left side of the patient and scope assistant towards client's head, without another first assistant

**Fig. 2:**
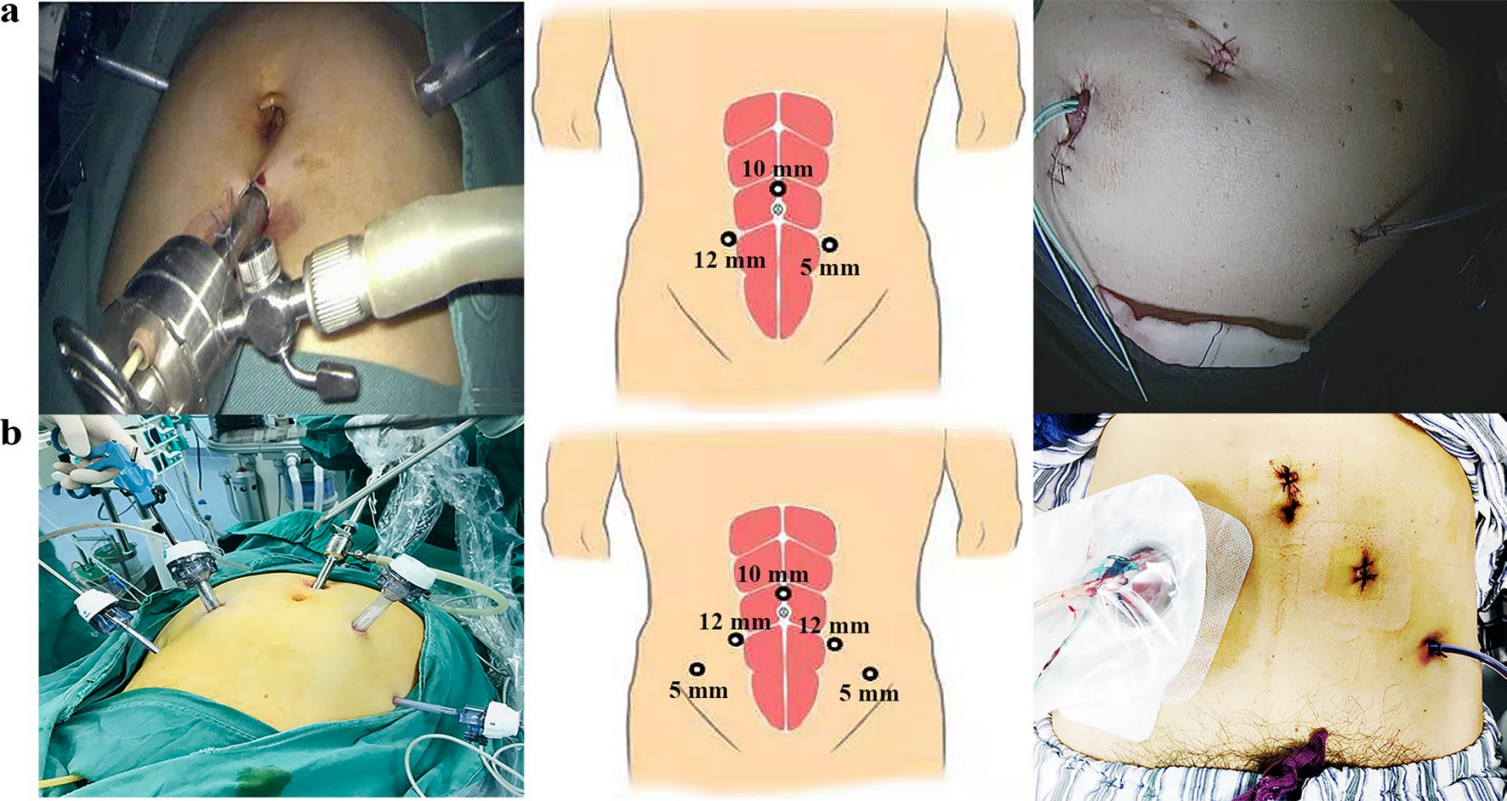
Position of the trocars and incisions for three-port LRC and five-port LRC. **a** Port placement and operative incision of three-port LRC with an ileal conduit; **b** Port placement and operative incision of five-port LRC with an ileal conduit

The three-port LRC technique was described in detail in our previous report [10]. In other words, the surgeon stood on the left side of the patient and a scope assistant was oriented toward the client's head, without the need for another first assistant (Fig. 1). The three-port approach was introduced: the first port (10-mm) was placed 2 cm above the umbilicus for the observation hole, followed by a hypogastric 12-mm port at the right lateral rectus line, 3–4 cm below the umbilicus for the main operation hole, and a 5-mm port for the assisted operation hole was placed at the left lateral rectus line, 5–7 cm below the umbilicus (Fig. 2a). Our surgical protocol mainly consists of five steps, with the approximate sequence as follows: dissociation of the ureter, dissociation of bladder and prostate ligaments, removal of the bladder and prostate, pelvic lymph node dissection (PLND), and extracorporeal construction of the ileal conduit. Our specific lateral three-layer approach was used to expose the vital pelvic anatomy, including the external iliac vessel layer, internal iliac vessel layer, and ureter layer. After completing RC and lymphatic dissection, pelvic re-peritonealisation was completed using Hem-o-lok clips (Teleflex Medical, Wayne, PA, USA). A conventional ileal conduit was constructed extracorporeally with a small midline incision. The ureters were implanted using a non-refluxing split-cuff nipple technique, which included a 0.5-cm longitudinal incision in the distal ureter, and the ureteral wall was turned back on itself to construct a nipple. Two F6 single-J stents were placed in the ureter to allow removal approximately 4 weeks postoperatively. The ureters were anastomosed to the ileal conduit in an end-to-side fashion with ureteral stents and secured with 4–0 absorbable suture. The ileal conduit was then placed into the peritoneal cavity, and the distal end of the ileal segment was anastomosed to the skin in a nipple-to-stoma fashion.

Conventional five-port LRC with an ileal conduit was performed in accordance with a previously described methom [9], including PLND, RC, and the use of a conventional ileal conduit. Five port placements were done in all patients (Fig. 2b). Pelvic re-peritonealisation and non-reflux techniques were used in the five-port group. All patients received standard postoperative care at our institution. All patients are encouraged to have early ambulation after surgery according to the principles of enhanced recovery after surgery (ERAS) [11] and gradually return to a regular diet after intestinal function recovery.

### Follow-up evaluation

All patients were followed-up in accordance with the institutional protocol. Follow-up data were obtained through teleconsultations, and outpatient services. Patients were evaluated at 1, 3, 6, and 12 months during the first year after surgery, and then semi-annually. Routine blood tests, renal function tests, urinary ultrasonography, or computed tomography were performed at every outpatient follow-up. The last follow-up time was August 2020.

### Data collection

All surgeries were completed by the same team. The data of patients were collected retrospectively, including demographic characteristics (e.g. age, sex, BMI, American Society of Anesthesiology [ASA] score, previous abdominal surgery, previous transurethral resection of bladder tumour [TURBT], smoking status, diabetes mellitus, hypertension, preoperative hydronephrosis, previous chemotherapy, and preoperative creatinine), operative and oncologic outcomes, and follow-up examination results. Complications that occurred within 90 days after surgery were defined as short-term complications, while those that developed 3 months after surgery were defined as long-term complications. Short-term complications (> 90 days) were categorised based on the Clavien-Dindo classification [12].

### Statistical analysis

All analyses were performed using SPSS 23.0 (IBM, Armonk, NY, USA). Results for continuous variables are expressed as mean ± standard deviations (SD) or median and interquartile range (IQR), and results for categorical variables are reported as the number of cases and percentages. To compare the differences between the two groups, continuous variables were assessed using Student’s t-test or the Mann–Whitney *U* test, and categorical variables were evaluated using the Chi-square test or Fisher’s exact test. All *P* values were two-sided, with *P* < 0.05 indicating statistical significance.

## Results

### Patient characteristics

The demographic characteristics of the two groups were shown in Table 1. We observed a more frequent history of previous abdominal surgery in the experimental group than in the control group (33.33% vs. 16.67%), although the difference was not statistically significant (*P* = 0.080). Thirteen patients had preoperative hydronephrosis (20.00% vs. 12.96% in the experimental and control groups, respectively, *P* = 0.399)*.* Therefore, there was no significant difference in the baseline characteristics between the two groups.

**Table 1:** Demographic characteristics of 84 bladder cancer patients

Variable	Total cases	Three-port LRC (n = 30)	Five-port LRC (n = 54)	*P* value
Age, [years, mean ± SD]	69.31 ± 9.36	70.84 ± 9.89	68.44 ± 9.03	0.258
Gender, [cases (%)]				0.093
Male	64 (76.2)	26 (86.50)	38 (70.40)	
Female	20 (23.8)	4 (13.50)	16 (29.60)	
Smoking status, [cases (%)]				0.090
Yes	40 (47.62)	18 (60.00)	22 (40.74)	
No	44 (52.38)	12 (40.00)	32 (59..26)	
Diabetes mellitus, [cases (%)]				1.00
Yes	14 (16.67)	5 (16.67)	9 (16.67)	
No	70 (83.33)	25 (83.33)	45 (83.33)	
Hypertension, [cases (%)]				0.461
Yes	32 (38.10)	13 (43.33)	19 (35.19)	
No	52 (61.90)	17 (56.67)	35 (64.81)	
Previous abdominal surgery*, [cases (%)]				0.080
Yes	19 (22.62)	10 (33.33)	9 (16.67)	
No	65 (77.38)	20 (66.67)	45 (83.33)	
Previous TURBT, [cases (%)]				0.973
Yes	31 (36.90)	11 (36.67)	20 (37.04)	
No	53 (63.10)	19 (63.33)	34 (62.96)	
Previous chemotherapy, [cases (%)]				0.398
Yes	26 (30.95)	11 (36.67)	15 (27.78)	
No	58 (59.05)	19 (63.33)	39 (72.22)	
BMI, [kg/m2,mean ± SD]	22.77 ± 3.03	22.36 ± 3.04	23.00 ± 3.03	0.359
ASA score, [cases (%)]				0.471
≤ 2	60 (71.43)	20 (66.67)	40 (74.07)	
> 2	24 (28.57)	10 (33.33)	14 (25.93)	
Preoperative hydronephrosis,[cases (%)]				0.399
Yes	13 (15.48)	6 (20.00)	7 (12.96)	
No	71 (84.52)	24 (80.00)	47 (87.04)	
Clinical tumour stage, [cases (%)]				0.609
≤ T1	28 (33.33)	8 (26.67)	20 (37.04)	
T2	32 (38.10)	13 (43.33)	19 (35.18)	
T3-4	24 (28.57)	9 (30.00)	15 (27.78)	
Preoperative creatinine, [umol/L, median (range)]	80 (49.00–143.50)	84 (39.00–143.50)	78 (44.00–135.00)	0.307

^*^History of abdominal surgery includes appendectomy, hernia operation, intestinal surgery and cesarean section

### Operative and pathological outcomes

**Fig. 3:**
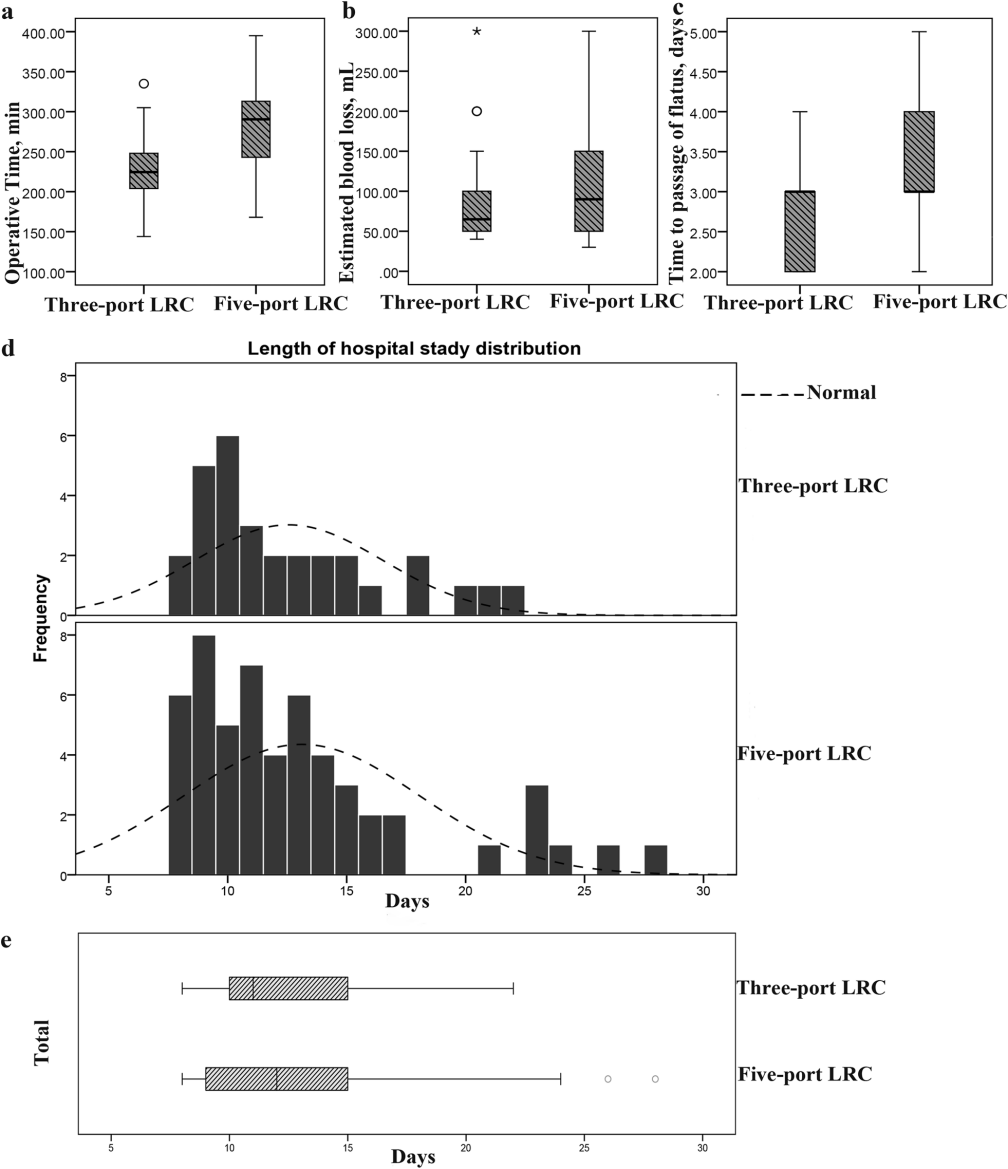
Comparison of perioperative results for three-port LRC and five-port LRC. **a** Comparison of operative time; **b** Comparison of estimated blood loss; **c** Comparison of time to passage of flatus; **d, e** Comparison of hospital stay after operation

The operative and pathological outcomes of patients in both groups are presented in Table 2. The operative time (271.30 ± 24.03 vs. 279.57 ± 48.47 min, *P* = 0.299) (Fig. 3a), estimated blood loss (65 vs. 90 mL, *P* = 0.352) (Fig. 3b), time to passage of flatus (8 vs. 10 days, *P* = 0.084) (Fig. 3c), and hospital stay after operation (11 vs. 12 days, *P* = 0.922) (Fig. 3d, e) were not significantly different between the two groups. Compared with the five-port group, the treatment cost was significantly lower in the three-port LRC group (12 453 vs. 14 134 $, *P* = 0.021). All cases had negative surgical margins, and patients with postoperative pathological results above T2b were administered adjuvant chemotherapy. Transitional cell carcinoma was the predominant type, seen in 83 (98.81%) cases, and squamous cell carcinoma was noted in the remaining cases (1.19%). In general, the surgical and pathological results did not show meaningful differences between these two groups, including pathology type, incidental prostate adenocarcinoma, tumour staging, and postoperative creatinine level (all *P* > 0.05). In addition, the results were similar in both groups in terms of postoperative recovery, such as time to ambulation after surgery, time to passage of flatus, time to regular diet, time to pelvic drain removal, and hospital stay after the operation (all *P* > 0.05).

**Table 2:** Operative and pathologic outcomes of 84 bladder cancer patients

Variable	Total cases	Three-port LRC (n = 30)	Five-port LRC (n = 54)	*P* value
Operative time, [min,mean ± SD]	276.62 ± 41.45	271.30 ± 24.03	279.57 ± 48.47	0.299
EBL, [mL,median (range)]	80 (30–300)	65 (40–300)	90 (30–300)	0.352
Pathology type, [cases (%)]				0.177
Transitional cell carcinoma	83 (98.81)	29 (96.67)	54 (100)	
Squamous cell carcinoma	1 (1.19)	1 (3.33)	0	
Incidental prostate adenocarcinoma,[cases(%)]	5 (5.95)	3 (10.00)	2 (3.70)	0.243
Pathologic T stage, [cases (%)]				0.166
1	30 (35.71)	6 (20.00)	24 (44.44)	
2	32(38.10)	14 (46.66)	18 (33.33)	
3	18 (21.43)	8 (26.67)	10 (18.52)	
4	4 (4.76)	2 (6.67)	2 (3.71)	
Pathologic N stage, [cases (%)]				0.563
0	75 (89.29)	26 (86.67)	49 (90.74)	
≥ 1	9 (10.71)	4 (13.33)	5 (9.26)	
Time to out-of-bed activity, [d, median (range)]	2 (1–4)	2 (1–4)	2 (1–4)	0.436
Time to passage of flatus, [d, median (range)]	3(2–5)	3(2–5)	3(2–5)	0.084
Time to regular diet, [d, median (range)]	5(4–13)	5(4–13)	6(4–12)	0.065
Time to pelvic drain removal, [d, median (range)]	9(5–18)	8(5–17)	10(5–18)	0.084
Hospital stay after operation, [d, median (range)]	11.5(7–28)	11(8–22)	12(7–28)	0.922
Median treatment cost [$, median (range)]*	13 474 (8 411–24 683)	12 453 (8 411–20 326)	14 134 (8 794–24 683)	0.021

^*^Treatment costs were converted to 2021 dollars

### Postoperative outcomes

The postoperative outcomes of these patients are shown in Table 3. Short-term complications were observed in 28 (33.33%) of 84 patients. Major complications (> grade III) were observed in three (10.00%) cases in the experimental group and in two (3.70%) in the control group (*P* = 0.174). In addition, two (6.67%) patients in the experimental group and one (1.85%) patient in the control group required reoperation due to severe bowel obstruction. However, our results indicated that the rate of short-term complications (43.33% vs. 27.78%, *P* = 0.274) and 90-day mortality (3.33% vs. 1.85%, *P* = 0.67) did not show meaningful differences between groups.

**Table 3:** Postoperative outcomes of 84 bladder cancer patients

Variable	Total cases	Three-port LRC (n = 30)	Five-port LRC (n = 54)	*P* value
Febrile urinary tract infection, [cases (%)]	11 (13.10)	6 (20.00)	5 (9.26)	0.162
Wound infection, [cases (%)]	5 (5.95)	2 (6.67)	3 (5.56)	0.837
Vein thrombosis, [cases (%)]	2 (2.38)	1 (3.33)	1 (1.85)	0.670
Bowel obstruction, [cases (%)]	6 (7.14)	2 (6.67)	4 (7.40)	0.899
Wound dehiscence, [cases (%)]	2 (2.38)	1 (3.33)	1 (1.85)	0.670
Sepsis, [cases (%)]	2 (2.38)	1 (3.33)	1 (1.85)	0.670
Reoperation, [cases (%)]	3 (3.57)	2 (6.67)	1 (1.85)	0.255
Clavien-Dindo classification (< 90d), [cases (%)]				0.274
I–II	23 (27.38)	10 (33.33)	13 (24.07)	
≥ III	5 (5.95)	3 (10.00)	2 (3.70)	
90-Days mortality, [cases (%)]	2 (2.38)	1 (3.33)	1 (1.85)	0.670

### Long-term outcomes

The median follow-up time was 17.5 months (range, 2–29 months) in the three-port group and 19 (range, 2–31) months in the five-port group (*P* = 0.433). Between 4–14 months after surgery, there were two (6.67%) cases of tumour recurrence in the three-port group and two (3.70%) cases in the five-port group (*P* = 0.541); they were undergoing chemoradiotherapy in the oncology department. At one month post-surgery, there were 16 (53.33%) cases of hydronephrosis in the three-port group and 27 (50.00%) cases in the five-port group. Twelve months after surgery, the three-port group had one (3.33%) case of severe hydronephrosis. In the five-port group, two (3.70%) patients with ureteroileal anastomotic stricture were diagnosed at 12 and 16 months after surgery, ipsilaterally in one patient and bilaterally in the other patient. One patient (1.85%) had a parastomal hernia at 10 months after surgery. These patients were kept under close surveillance, and the complications gradually resolved with surgical intervention.

## Discussion

This study aimed to compare the clinical outcomes between the three-port approach and conventional five-port LRC for bladder cancer. Our results confirmed the feasibility of three-port LRC with an ileal conduit in treating invasive bladder cancer, with similar perioperative outcomes and complication rates as the standard five-port LRC, but the treatment costs of the former were significantly less.

ORC is the primary surgical modality for muscular-invasive bladder cancer or high-risk non-muscular-invasive bladder cancer, with a perioperative complication rate of up to 60% due to the complexity of the procedure [13]. With the rapid development of minimally invasive technology, RARC and LRC have become important procedures for bladder cancer management, having been carried out in several medical centres [2]. Numerous studies have demonstrated the advantages of these procedures in reducing the rate of complications and accelerating postoperative recovery, and their safety and tumour therapeutic efficacy are similar to those of ORC [5, 13, 14]. Therefore, they have been used as one of the first-line surgical treatments of bladder cancer in some developed countries. Nevertheless, the complex operation of the robotic system extends the operation time, and the huge costs are not negligible. Morii et al. [15] found that RARC is more expensive than LRC and ORC. In contrast, LRC is widely accepted because of its lower cost and satisfactory efficacy, especially in developing countries such as China. This might not change for a long time. Therefore, traditional LRC remains vital in these countries and has become more prevalent with the increasing incidence of bladder cancer. In fact, owing to the financial cost of RARC [16], standard LRCs are still used in some large medical centres.

For a variety of reasons, urologists in our centre mainly perform LRC with different urinary diversions. Common trocar placement mostly involve the four-port or five-port method in conventional LRC, which is mainly performed by three or four urologists. Although LRC has become quite popular, some limitations have yet to be overcome, such as complex operation, the unfamiliar coordination of the surgeon and assistant, parietal pain, and high operating costs [2, 3, 15]. Recently, reduced port surgery has become increasingly popular for LRC. Single-port and two-port LRC have been reported as minimally invasive methods with potential advantages in port site-related complications, improved cosmesis, and fewer surgical incisions [17, 18]. Moreover, the operation was completed by a urologist and a scope assistant by performing a few incisions. However, these methods have several disadvantages compared with conventional laparoscopic surgery, including long operation time, difficulty in patients with obesity or narrow pelvic space, and a steep learning curve. To overcome the above limitations and the lack of effective coordination due to the operator's and assistant's unfamiliarity with the four-port or five-port LRC, we also attempted to reduce the ports in LRC based on previous surgical experience and performed the three-port procedures for bladder cancer. Our initial goal was to simplify the procedure, make the incision ideal, and facilitate patient recovery [10]. In contrast, the three-port procedure is not substantially different from the single-port and two-port methods and is not restricted by the first assistant compared with the conventional five-port procedure.

Although most of the recent single-centre reports are experiences with RARC and LRC, the availability and cost of technology are major concerns [3, 5, 6]. In our view, three-port LRC has the advantage of an acceptable minimally invasive incision and low cost. Our results show that three-port LRC is not significantly different from standard LRC in oncological results and operative time, but the treatment costs of the former are significantly less. Although inpatient costs are associated with multiple factors, such as the commodity price and health insurance policies, the reduction in the number of surgeons and instruments can avail economic benefits.

Laparoscopic surgery has become quite popular in urology and has potential advantages in precision surgery and tumour control [19]. We also note further modifications of traditional laparoscopic techniques, including the use of the three-port method, in prostate cancer patients. Xu et al. [20] believe that the three-port method has more advantages than traditional laparoscopic radical prostatectomy, including surgical results and postoperative complications. The main benefits of the three-port approach include: (1) fewer surgical incisions and low treatment costs, (2) overcomes the influence of assistants and main equipment, and (3) potential advantages of triangulation. Similarly, our three-port method, which is based on the traditional five-port LRC, overcomes the limitations of the first assistant and the main surgical equipment, which poses significant disadvantages when implementing a five-port technique. Urologists can use left-handed instruments to separate the surrounding tissues and expand the surgical field even for patients with obesity, narrow pelvic organ space, and factors leading to difficulty in exposure. Triangulation is an important basic principle of laparoscopic surgery, which can avoid the formation of narrow spaces and reduce the surgeon's fatigue. The triangular trocar distribution is consistent with the ergonomic principle, which is also in line with the surgeon's habit of laparoscopic surgery. Combined with other urologists’ experience in Table 4, we observed that three-port LRC may have an advantage in shortening operative time. It is worth noting that with the reduction in the number of trocars, the three-port approach requires more experience and ability for surgeons. However, our method is difficult to be used by beginners. Therefore, this is recommended for surgeons with extensive experience in laparoscopic surgery.

**Table 4:** A synopsis of published series on the surgical treatment of bladder cancer

Reference	Treatment	Case(n)	OT(min)	EBL (ml)	Hospital stay after operation (days)	Complications (%)	PSM (%)
Huang et al. [21]	Single-port LRC with orthotopic ileal neobladder	8	399	154	15	37.50	NA
Ma et al. [18]	Single-port LRC with ileal conduit	5	208.02^a^ /135^b^	270	19.5	20.00	0
Angulo et al. [17]	Two-port LRC with urinary diversion	30	330	347.5	10	40.00	0.067
Abraham et al. [22]	Conventional LRC with ileal conduit	20	419	653	9.4	70.00	0
Khan et al. [23]	Conventional LRC with urinary diversion	58	316	480.7	16.1	27.00	0.04
Kim et al. [24]	Conventional LRC with urinary diversion	22	524	400	12	NA	0
Su et al. [25]	Conventional LRC with urinary diversion	126	315	200	10	58.73	0.023

^a^Extirpative operative time; ^b^ileal conduit operative time

The Bricker ileal conduit remains the classic and standardized procedure for urinary diversion following RC, widely used by urologists due to its relatively simple procedure and low postoperative complication rate [26]. In our study, all patients underwent a Bricker ileal conduit to reduce the impact of different urinary diversions on postoperative outcomes. Our follow-up results showed that there were no significant differences between the two groups, including short-term and long-term complications. Thus, these results indicate that the three-port LRC with an ileal conduit is feasible for the treatment of bladder cancer. It is important to note that anastomotic techniques may affect the rate of postoperative complications after an ileal conduit, and controversies surrounding refluxing or nonrefluxing anastomotic techniques remain. Nonrefluxing techniques have a higher rate of strictures than the refluxing technique, but the latter has harmful effects on renal function because of potential urinary infection [27, 28]. However, some researchers believe that these techniques have no significant effect on long-term outcomes [29]. In this study, our team performed a split-cuff ureteric nipple to avoid urine reflux according to previous experiences. Our clinical data showed that the rate of postoperative hydronephrosis in the control group was higher than that in the study group, but the difference was not statistically significant. The hydronephrosis in the two groups, which may be related to inflammatory oedema at the anastomotic site, gradually resolved with time after surgery. It is important to protect renal function after an ileal conduit because upper urinary tract obstruction from a stricture is a major cause of renal damage [30]. In our follow-up, these patients had no overtly impaired renal function, and only two patients in the control developed strictures. However, the occurrence of late postoperative strictures requires further investigation.

This study has several limitations that need to be addressed. First, as this was a small-sample retrospective study, randomised controlled trials were not performed and a contemporary historical cohort was used as a control group for comparison. Second, the follow-up time of this study was relatively short, which can fail to determine all complications of the technique, especially long-term complications. Further studies are needed to confirm the clinical relevance of these findings.

## Conclusions

In our study, we observed that the perioperative outcomes and complications did not show any significant difference between the three-port LRC with an ileal conduit and a conventional five-port LRC with an ileal conduit, but the treatment costs of the former were reduced. These preliminary data suggest that our three-port LRC may be a viable alternative LRC method. However, further large-sample prospective randomised controlled trials are required to confirm the benefits and long-term effects of the three-port approach.

## Abbreviations

LRC: Laparoscopic radical cystectomy
RC: Radical cystectomy
ORC: Open radical cystectomy
LRC: Laparoscopic radical cystectomy
RARC: Robot-assisted radical cystectomy
BMI: Body mass index
PLND: Pelvic lymph node dissection
SD: Standard deviations
IQR: Interquartile range
ASA: American society of anesthesiology
TURBT: Transurethral resection of bladder tumour
ERAS: Enhanced recovery after surgery
OT: Operative time
EBL: Estimated blood loss
PSM: Positive surgical margin
NA: Not available

## Declarations

### Availability of data and materials

The datasets used and/or analysed during the current study are available from the corresponding author on reasonable request.
